# Supplementary material for: Real-time motor operating state recognition via multi-sensor fusion: A wavelet–neural–evidence framework for industrial condition monitoring
Source: PLoS One. 2025 Nov 7;20(11):e0335360. doi: 10.1371/journal.pone.0335360 (PMC12594399; doi:10.1371/journal.pone.0335360)
Supplement: S1 Text — (PDF) [file pone.0335360.s001.pdf]

%The main procedure of this article is as follows. Please contact the correspondent if it is complete.

File structure

```
motor_monitoring/
 ├── main_motor_monitoring.m
 ├── preprocess_signal.m
 ├── extract_wavelet_features.m
 ├── train_bp_network.m
 ├── ds_fusion.m
 ├── evaluate_results.m
 ├── utils/
 │    ├── plot_confidence_distribution.m
 │    ├── plot_confusion_matrix.m
 │    └── plot_roc_curves.m
```

```
% Main script for motor condition monitoring using multi-sensor fusion

clear; clc; close all;

% Load data (simulated or experimental)
```

```
load('data/motor_signals.mat'); % Assumed format: struct with vibration, acoustic, magnetic

% Step 1: Preprocess and extract features
features = preprocess_signal(raw_data);

% Step 2: Train BP neural networks for each sensor modality
[model_vib, pred_vib] = train_bp_network(features.vibration, labels);
[model_acou, pred_acou] = train_bp_network(features.acoustic, labels);
[model_mag, pred_mag] = train_bp_network(features.magnetic, labels);

% Step 3: Perform Dempster-Shafer fusion
fused_result = ds_fusion({pred_vib, pred_acou, pred_mag});

% Step 4: Evaluate and visualize
evaluate_results(fused_result, labels);
```

```
function features = preprocess_signal(raw_data)
% Perform wavelet packet decomposition and feature extraction

features.vibration = extract_wavelet_features(raw_data.vibration, 3);
features.acoustic = extract_wavelet_features(raw_data.acoustic, 3);
features.magnetic = extract_wavelet_features(raw_data.magnetic, 3);
end

function feat = extract_wavelet_features(signal, level)
% Apply wavelet packet decomposition and compute relative energy features

n = size(signal, 1);
feat = zeros(n, 8); % Assuming level-3 => 8 subbands

for i = 1:n
    wp = wpdec(signal(i,:), level, 'db4');
    total_energy = 0;
```

```
        energies = zeros(1, 8);
        for j = 0:7
            node = wpfind(wp, 'type', 'terminal', 'depth', level);
            coef = wprcoef(wp, node(j+1));
            energies(j+1) = sum(coef.^2);
            total_energy = total_energy + energies(j+1);
        end
        feat(i, :) = energies / total_energy;
    end
end

function [net, predictions] = train_bp_network(features, labels)
% Train a 3-layer BP network and return softmax predictions

targets = full(ind2vec(labels'+1)); % One-hot encoding
net = patternnet([10, 10]); % Two hidden layers
net.trainParam.showWindow = false;
net.trainParam.epochs = 300;
net = train(net, features', targets);
```

```
predictions = net(features')';
end

function fused = ds_fusion(pred_list)
% Perform Dempster-Shafer fusion on multiple softmax outputs

n = size(pred_list{1}, 1);
num_class = size(pred_list{1}, 2);
fused = zeros(n, num_class);

for i = 1:n
    m = pred_list{1}(i, :);
    for j = 2:length(pred_list)
        m2 = pred_list{j}(i, :);
        K = sum(m .* m2 .* (1 - eye(num_class)));
        m_comb = (m .* m2) / (1 - K + eps);
        fused(i, :) = m_comb / sum(m_comb);
        m = fused(i, :);
    end
end
end
```

```
function evaluate_results(pred, labels)
% Evaluate prediction accuracy and plot relevant metrics

[~, y_pred] = max(pred, [], 2);
acc = mean(y_pred == labels);
fprintf('Fusion Accuracy: %.2f%%\n', acc*100);

% Plotting
plot_confidence_distribution(pred);
plot_confusion_matrix(y_pred, labels);
plot_roc_curves(pred, labels);
end

clc; clear;

% Load multi-sensor dataset
load('motor_dataset.mat'); % Assume data contains vibration, acoustic, magnetic, and labels

% Parameters
```

```
fs = 512;                               % Sampling frequency
window_length = 1024;         % 2 seconds
overlap = 0.5;                        % 50% overlap
decomp_level = 3;                 % WPT level

% Segment data and extract features
[E_vib, E_mag, E_ac, labels] = extract_all_features(vibration, magnetic, acoustic, gt_labels, fs, window_length, overlap, decomp_level);

% Train BP networks per channel
net_vib = train_bp(E_vib, labels);
net_mag = train_bp(E_mag, labels);
net_ac    = train_bp(E_ac, labels);

% Predict softmax outputs
y_vib = net_vib(E_vib')';
y_mag = net_mag(E_mag')';
y_ac    = net_ac(E_ac')';

% D-S fusion
fused_label = ds_fusion(y_vib, y_mag, y_ac);
```

```
% Evaluate performance
evaluate_results(labels, fused_label, y_vib, y_mag, y_ac);
function [E_vib, E_mag, E_ac, Y] = extract_all_features(vib, mag, ac, labels, fs, win_len, overlap, level)
        step = win_len * (1 - overlap);
        num_win = floor((length(labels) - win_len) / step);

        E_vib = []; E_mag = []; E_ac = []; Y = [];

        for i = 1:num_win
            idx = round((i-1)*step + 1);
            v_seg = vib(idx:idx+win_len-1,:);
            m_seg = mag(idx:idx+win_len-1,:);
            a_seg = ac(idx:idx+win_len-1,:);
            lb = mode(labels(idx:idx+win_len-1));

            E_v = extract_wpt_energy(v_seg, level);
            E_m = extract_wpt_energy(m_seg, level);
```

```
        E_a = extract_wpt_energy(a_seg, level);

        E_vib = [E_vib; E_v];
        E_mag = [E_mag; E_m];
        E_ac    = [E_ac;    E_a];
        Y = [Y; lb];
    end
end

function y_final = ds_fusion(y1, y2, y3)
    N = size(y1,1);
    y_final = zeros(N,1);
    for i = 1:N
        m1 = y1(i,:);
        m2 = y2(i,:);
        m3 = y3(i,:);
        mf = ds_comb(ds_comb(m1, m2), m3);
        [~, y_final(i)] = max(mf);
        y_final(i) = y_final(i) - 1;
    end
end
```

```
function m = ds_comb(m1, m2)
    K = sum((m1'*ones(1,length(m2))) .* (ones(length(m1),1)*m2), 'all') - sum(m1 .* m2);
    m = (m1 .* m2) / (1 - K + eps);
end

function evaluate_results(gt, pred, y1, y2, y3)
    acc = sum(gt == pred) / length(gt);
    fprintf('D-S Fusion Accuracy: %.2f%%\n', acc*100);

    % Confusion matrix
    figure;
    confusionchart(gt, pred);
    title('Confusion Matrix of D-S Fusion Model');

    % ROC for each class and each channel
    classes = unique(gt);
    for i = 1:length(classes)
        subplot(1,3,i);
        [~,~,~,auc1] = perfcurve(gt, y1(:,i), classes(i));
```

```
        [~,~,~,auc2] = perfcurve(gt, y2(:,i), classes(i));
        [~,~,~,auc3] = perfcurve(gt, y3(:,i), classes(i));
        plot([auc1 auc2 auc3], '-o');
        legend('Vibration','Magnetic','Acoustic');
        title(['AUC for Class ', num2str(classes(i))]);
    end
end
```
