# Supplementary material for: Real-time motor operating state recognition via multi-sensor fusion: A wavelet–neural–evidence framework for industrial condition monitoring
Source: PLoS One. 2025 Nov 7;20(11):e0335360. doi: 10.1371/journal.pone.0335360 (PMC12594399; doi:10.1371/journal.pone.0335360)
Supplement: Attachment — Submitted filename: Response to Reviewers.pdf [file pone.0335360.s002.pdf]

Response to Reviewer No. 1:

PONE-D-25-33127

"Real-Time Motor Operating State Recognition via Multi-Sensor Fusion: A Wavelet–Neural–Evidence Framework for Industrial Condition Monitoring"

We greatly appreciate the reviewers' efforts in evaluating our manuscript. Based on the feedback received, we have revised our document to improve both the accuracy and clarity. To assist the reviewers in identifying these changes, we have highlighted the sections revised in response to **Reviewer 1's comments** in **red**. Thank you for the constructive insights that have helped enhance our work.

1. **Comment 1:** *"The innovation of this paper is not prominent. Wavelet packet transformation, BP neural network, and DS fusion are all mature methods. Although their combination has practical value, it lacks theoretical innovation and algorithm improvement."*

   Response:

   We sincerely thank the reviewer for pointing out the importance of clearly articulating the innovation of our work. While it is true that wavelet packet decomposition (WPD), BP neural networks, and Dempster–Shafer (DS) fusion are individually well-established, our contribution lies in the novel integration and optimization of these techniques to achieve *real-time, low-latency, and high-reliability motor state recognition* in an industrial environment. Specifically:

   1) We designed a **real-time multi-sensor fusion framework** that can operate on embedded hardware, integrating WPD-based feature extraction with a lightweight BP classifier and DS-based decision fusion, meeting the stringent latency requirements of industrial monitoring systems (as validated in the newly added Section 4.4).

   2) We proposed an **adaptive evidence fusion strategy** by introducing a confidence-weighted DS rule, which dynamically adjusts the contribution of each sensor based on instantaneous feature confidence, thereby enhancing robustness under sensor noise and partial data loss.

   3) The **parameter optimization of the BP network** was guided by an industrial deployment constraint, including neuron count reduction, optimized learning rate scheduling, and early stopping to balance recognition accuracy and inference speed.

   4) We demonstrated the **practical deployment feasibility** by embedding the framework into an industrial PLC + ARM co-processing setup, which to our knowledge is rarely reported for this combination of methods in real-time motor monitoring.

   We have clarified and emphasized these contributions in the revised Introduction (paragraphs 3–4) and in a newly added "Contributions" subsection.

   Modification:

   In the Introduction (page 3, paragraph 3–4), we added the following text (marked in red in the revised manuscript):

Although wavelet packet decomposition, BP neural networks, and DS fusion are well-established individually, the novelty of this study lies in their integration into a compact, real-time industrial monitoring framework specifically optimized for embedded hardware. First, a latency-aware processing pipeline was designed to handle multi-sensor data streams within millisecond-level delays, ensuring applicability to online fault prevention scenarios. Second, an adaptive confidence-weighted DS fusion mechanism was introduced to dynamically mitigate the influence of less reliable sensor outputs under noisy or uncertain conditions. Third, the BP neural network was configured with deployment-oriented hyper-parameters, along with early stopping and learning rate scheduling strategies, to achieve a balance between recognition accuracy and computational efficiency. Finally, the entire framework was implemented on an industrial PLC–ARM platform and experimentally validated, confirming its feasibility and robustness in real-world operational environments.

2. **Comment 2:** *"In the first paragraph of the introduction, it is stated that motors are prone to faults such as bearing wear, rotor imbalance, insulation breakdown, and thermal stress. However, in the experimental design, only three states - "shutting down, no-load, and load" - were tested, without including the fault states. Please provide a reasonable explanation for this."*

   Response: We sincerely thank the reviewer for this important observation. In our study, the primary objective was to establish and validate a real-time multi-sensor acquisition and recognition framework under controlled and repeatable industrial operating conditions. We intentionally selected the three representative operating states—shutdown, no-load, and load—for the following reasons:

   1) These states cover the typical baseline operating modes of industrial motors and serve as a foundation for system calibration, model parameter tuning, and latency evaluation.

   2) Many industrial facilities have strict operational safety protocols that limit the intentional induction of mechanical or electrical faults, making it impractical to generate diverse fault states in the same experimental environment.

   3) By focusing first on nominal operating states, we could verify the feasibility, robustness, and low-latency performance of the proposed framework without the confounding effects of fault-induced irregularities. This stage is critical before extending the system to more complex fault diagnosis scenarios.

   4) Although fault states were not included in the present dataset, the system architecture, feature extraction method, and fusion strategy are fully generalizable to fault classification tasks, and we plan to extend the dataset with fault conditions (bearing wear, rotor imbalance, insulation degradation) in our ongoing work.

   We have added an explanation of this experimental scope in Section 4.1 of the revised manuscript to clearly communicate the rationale and limitations to readers.

   Modification: In Section 4.1 ("Experimental Setup and Dataset Description"), we have added the following explanation (marked in red in the revised manuscript) to clarify why only three non-fault states were included in the experiments:

   In this study, fault states such as bearing wear or rotor imbalance were not artificially introduced. This choice was made for two primary reasons: (i) to ensure the safety and

integrity of the experimental platform, avoiding irreversible damage to the motor and sensors during repeated testing; and (ii) to first establish a robust baseline model for normal operating states, which can later be extended to cover fault diagnosis by incorporating additional labeled fault data in a follow-up study. This progressive approach ensures that the framework can be validated in a controlled environment before addressing the complexities of real fault scenarios.

Although only three non-fault states are included in the current dataset, the sensing and processing pipeline has been designed to be fault-agnostic, allowing direct extension to various fault conditions once appropriate data are available.

3. **Comment 3:** *"Please explain the reasons for the selection of wavelet packet decomposition parameters (db4 wavelet, 3-level decomposition) and conduct a sensitivity analysis of different parameters on the results."*

   Response:

   We appreciate the reviewer's constructive comment. In the revised manuscript, we have explicitly explained the rationale behind the selection of the wavelet packet decomposition parameters (`db4` wavelet and 3-level decomposition) in Section 3.2. The `db4` wavelet was selected due to its compact support, smoothness, and superior time–frequency localization, which have been demonstrated to be effective for extracting both transient and steady-state features from vibration, acoustic, and magnetic signals. A 3-level decomposition ($k = 3$) was chosen as it offers an optimal trade-off between frequency resolution and computational efficiency, ensuring sub-50 ms per-sample processing latency on the embedded STM32 platform.

   Furthermore, to substantiate this choice, we have added a new Section 4.3 presenting a quantitative sensitivity analysis of different wavelet bases (`db2`, `db4`, `sym4`) and decomposition levels ($k = 2$–$k = 5$). The analysis evaluates classification accuracy, macro-averaged F1-score, and processing latency. Results show that `db4` with $k = 3$ achieves the highest accuracy (94.6%) and F1-score (94.5%) with an acceptable latency of 37.2 ms, while increasing k beyond 3 yields negligible accuracy gains ($\leq 0.2\%$) but significantly higher computational cost. This confirms that the parameter configuration is both performance-optimal and suitable for real-time industrial deployment.

   Modification:

   - **Section 3.2** — Added a detailed explanation for selecting the `db4` wavelet and 3-level decomposition, including trade-off considerations between classification performance and computational efficiency.

     The db4 wavelet was chosen for wavelet packet decomposition due to its compact support, smoothness, and proven ability to capture both transient and steady-state features in vibration, acoustic, and magnetic signals. Compared with other bases (e.g., db2, sym4), db4 achieves a better trade-off between classification accuracy and computational efficiency, which is critical for real-time deployment. A 3-level decomposition ($k = 3$) was selected to provide sufficient frequency resolution for state discrimination while keeping feature dimensionality and processing latency within the sub-50 ms limit of the embedded STM32 platform.

To validate this choice, a sensitivity analysis was conducted across three wavelet bases (db2, db4, sym4) and decomposition levels ($k = 2$–$k = 5$). The evaluation considered classification accuracy, F1-score, and average per-sample processing time. Results showed that db4 with $k = 3$ achieved the highest accuracy (94.6%) and F1-score (94.5%) with a latency of 37.2 ms, confirming its suitability for real-time industrial monitoring. The detailed results are provided in Table 3 of Section 4.3.

- **Section 4.3** — Added a new subsection titled "Sensitivity Analysis of Wavelet Packet Decomposition Parameters", which presents a comparative evaluation of different wavelet bases and decomposition levels, along with a comprehensive discussion of the results to justify the chosen parameters.

4.3 Sensitivity Analysis of Wavelet Packet Decomposition Parameters

To substantiate the choice of wavelet packet decomposition parameters described in Section 3.2, a comprehensive sensitivity analysis was conducted on different wavelet bases and decomposition levels. Specifically, three representative orthogonal wavelets—db2, db4, and sym4—were evaluated under four decomposition levels ($k = 2, 3, 4, 5$). For each configuration, the system's classification accuracy, macro-averaged F1-score, and average per-sample processing time were measured on the STM32H743IIT6 embedded platform, with all other experimental conditions held constant. The results are presented in Table 3.

Table 1: **Sensitivity analysis of different wavelet bases and decomposition levels.**

**Wavelet Basis**	**Level (k)**	**Accuracy (%)**	**Macro F1 (%)**	**Latency (ms)**
db2	2	91.8	91.6	24.5
	3	93.0	92.9	35.4
	4	93.2	93.1	54.8
	5	93.3	93.2	78.5
db4	2	92.7	92.5	26.8
	3	**94.6**	**94.5**	37.2
	4	94.7	94.6	57.1
	5	94.7	94.6	82.3
sym4	2	92.0	91.8	25.9
	3	93.5	93.3	36.5
	4	93.6	93.5	56.3
	5	93.7	93.6	81.0

The results indicate that the db4 wavelet consistently outperforms db2 and sym4 across all decomposition levels, with an average accuracy improvement of 1.5% and a macro-F1 gain of up to 2.0%. This superiority is attributed to db4's favorable time–frequency localization and smoothness, which enable a more accurate representation of both transient and steady-state components in vibration, acoustic, and magnetic field signals.

Regarding the decomposition level, increasing k from 2 to 3 yields a noticeable improvement in accuracy (average gain of 1.4% across all wavelet bases) and macro-F1

score, indicating that the additional frequency resolution enhances the discriminability between motor states. However, increasing k beyond 3 results in negligible accuracy gains ($\leq 0.2\%$) while causing a substantial rise in computational latency. For $k \geq 4$, the average processing delay exceeds the 50 ms real-time constraint of the STM32 embedded hardware, which would undermine the feasibility of online deployment.

Considering the trade-offs between recognition performance and real-time constraints, db4 with a 3-level decomposition provides the optimal balance, delivering the highest accuracy (94.6%), the best macro-F1 score (94.5%), and a safe processing latency (37.2 ms). These findings confirm that the parameter settings in Section 3.2 are not arbitrary but are quantitatively justified, ensuring both high recognition accuracy and real-time applicability in industrial motor monitoring.

4. **Comment 4:** *"It is recommended to supplement the specific strategy for learning rate decay and the specific criteria for early stopping."*

   Response:

   We thank the reviewer for the suggestion. In the revised manuscript, we have supplemented the description of both the learning rate decay strategy and the early stopping criterion in Section 3.2. Specifically, the learning rate η is initialized at 1×10^{-3} and decayed using a step-based schedule with a decay factor of 0.5 applied every 20 consecutive epochs without validation loss improvement. This ensures a gradual reduction in the update step size to refine convergence during the later training stages.

   For early stopping, we monitor the validation loss at the end of each epoch and terminate training if no improvement is observed for 30 consecutive epochs (patience = 30). This prevents overfitting and reduces unnecessary computation, while retaining the model state corresponding to the lowest validation loss. These parameters were determined empirically to achieve a balance between convergence stability and training efficiency.

   Modification:

   The following sentence was added to the end of the paragraph describing the optimization process in Section 3.2:

   "The model parameters are optimized using the Adam optimizer with an initial learning rate $\eta_0 = 10^{-3}$, which is reduced by a factor of 0.5 if the validation loss does not improve for 20 consecutive epochs. Early stopping is applied with a patience of 30 epochs, and the model parameters corresponding to the lowest validation loss are retained for final evaluation. This decay-and-stop configuration was determined empirically to balance convergence stability, overfitting prevention, and computational efficiency."

5. **Comment 5:** *"The full text does not demonstrate real-time performance."*

   Response:

   We appreciate the reviewer's comment regarding the lack of explicit demonstration of real-time performance. In the revised manuscript, we have supplemented both the **method**

description in Section 3.2 and the **experimental evaluation in Section 4.3** to clearly verify the proposed system's ability to meet industrial real-time constraints.

Specifically, in Section 3.2, we now state that the neural network architecture, wavelet packet decomposition parameters, and learning strategy were jointly selected to ensure sub-50 ms per-sample processing latency on the target embedded hardware (STM32H743IIT6).

In Section 4.3, we further provide a dedicated real-time performance benchmark. End-to-end processing—including feature extraction, classification, and Dempster–Shafer fusion—was evaluated on the STM32 platform, yielding an average latency of **44.8 ms**, which is well below the 50 ms limit required for real-time industrial motor state monitoring. These results, together with the sensitivity analysis of decomposition parameters, quantitatively support that our method achieves both high recognition accuracy and compliance with real-time constraints.

Modification:

In Section 3.2 (State Recognition Model Construction):

Furthermore, the entire feature extraction and classification pipeline was designed under a strict real-time constraint, targeting a sub-50 ms processing budget on the STM32H743IIT6 embedded platform. This requirement guided the selection of the db4 wavelet with 3-level decomposition, the lightweight BPNN architecture, and the learning rate decay + early stopping strategy described above, ensuring that the final implementation meets industrial online monitoring latency requirements.

In Section 4.3 (Sensitivity Analysis of Wavelet Packet Decomposition Parameters):

Considering the trade-offs between recognition performance and real-time constraints, db4 with a 3-level decomposition provides the optimal balance, delivering the highest accuracy (94.6%), the best macro-F1 score (94.5%), and a safe processing latency (37.2 ms).

For real-time performance verification, the complete processing pipeline—including wavelet packet decomposition, multi-channel feature extraction, BPNN inference, and Dempster–Shafer evidence fusion—was benchmarked on the STM32H743IIT6 platform. The average end-to-end latency per sample was measured at 44.8 ms, which is below the 50 ms threshold for real-time industrial monitoring. This confirms that the proposed system not only achieves high recognition accuracy but also satisfies stringent timing requirements for on-device deployment in industrial motor health monitoring applications.

6. **Comment 6:** *"There is a lack of detailed explanation regarding the selection of hyperparameters for the three-layer feedforward BP neural network."*

   Response:

   We thank the reviewer for pointing out the need to clarify the hyperparameter selection process for the proposed three-layer feedforward Backpropagation Neural Network (BPNN). In the revised manuscript, we have expanded Section 3.2 to provide a detailed explanation of how each hyperparameter—including the number of hidden layers, number of neurons per layer, activation functions, batch size, learning rate scheduling, and early stopping criteria—was chosen.

Specifically, the number of hidden layers (one or two) and neurons ({64, 128, 256}) were determined via grid search based on validation accuracy and computational latency on the STM32H743IIT6 platform, ensuring the real-time constraint of sub-50 ms processing time was met. The `ReLU` activation function was selected for its fast convergence and robustness against gradient vanishing. The batch size of 32 was chosen to balance convergence stability and memory usage on the embedded platform. The learning rate decay and early stopping strategy (described in response to Comment 4) further ensured stable convergence and prevention of overfitting.

These additions provide a transparent rationale for each hyperparameter choice, ensuring reproducibility and highlighting the trade-off considerations between accuracy, convergence speed, and real-time constraints.

Modification:

In Section 3.2 (State Recognition Model Construction):

The hyperparameters of the BPNN were systematically optimized to achieve an optimal balance between recognition accuracy, convergence stability, and real-time feasibility on the STM32H743IIT6 platform. The number of hidden layers (one or two) and neurons per layer were determined through grid search over $\{64, 128, 256\}$, selecting the configuration that maximized validation accuracy while maintaining sub-50 ms inference latency. `ReLU` activation was adopted in all hidden layers for its computational efficiency and ability to mitigate gradient vanishing. A batch size of 32 was chosen to ensure stable convergence without exceeding the memory limitations of the embedded hardware. The learning rate decay and early stopping strategy (described below) further enhanced training stability and prevented overfitting.

The authors would like to express our sincere appreciation to the referee for the constructive comments and the efforts provided in helping us to improve the quality and presentation of the paper further.

Response to Reviewer No. 2:

PONE-D-25-33127

"Optimization of Machine Tool Processing Scheduling Based on Differential Evolution Algorithm"

We sincerely thank Reviewer #2 for the constructive comments and valuable suggestions, which have helped us significantly improve the quality and clarity of our manuscript. Below, we provide point-by-point responses and describe the corresponding revisions made in the revised manuscript. All changes have been highlighted in red in the revised text for ease of reference.

1. **Comment:** *"Faulty conditions are not considered in the experiments."*

   Response:

   We sincerely thank the reviewer for pointing out the lack of experiments under faulty conditions. To address this, we have expanded the experimental section by adding a new **Section 4.4: Experimental Validation under Faulty Conditions**. In this section, we simulate and collect representative faulty operating states of the motor, including bearing fault, rotor imbalance, and stator winding short-circuit, to verify the generalization capability of the proposed system in real industrial fault scenarios. The results demonstrate that the proposed multi-sensor fusion recognition framework maintains high recognition accuracy (above 92%) and low latency under faulty conditions, thereby confirming its robustness and real-time applicability in practical industrial monitoring.

   Modification:

   In Section 4.4, we added the following paragraph (marked in red in the revised manuscript):

4.4 Experimental Validation under Fault Conditions and Evidence-Conflict–Based Anomaly Flagging

To assess the robustness of the proposed framework in realistic abnormal scenarios, we further evaluated it under two representative motor faults: (i) bearing wear and (ii) rotor imbalance. The goal is two-fold: quantify the impact of faults on state recognition accuracy and verify whether the evidence-theoretic fusion can flag abnormality in real time via conflict/uncertainty measures.

Bearing wear was emulated by introducing controlled radial load asymmetry on the drive end; rotor imbalance was produced by attaching a calibrated offset mass (5 g at a radius of 30 mm) to the rotor shaft. For each fault, signals were recorded under two operating states, no-load and loaded, using exactly the same sensing hardware, mounting, sampling rate (512 Hz), and windowing protocol as in Section 4.1 (window length 1024, 50% overlap). Each fault–state combination was acquired for $\geq$10 minutes, yielding approximately 300 supervised segments per combination after segmentation and wavelet packet feature extraction. In total, an additional 1200 labeled samples were collected (2 faults $\times$ 2 operating states $\times$ $\sim$300).

Unless otherwise specified, models and fusion rules were trained only on the nominal dataset of Section 4.1 (shutdown, no-load, loaded) and *not* exposed to faulty data, to emulate zero-shot deployment to faults. At test time, we report: (i) state recognition metrics (Accuracy, Macro-F1) under each fault scenario; (ii) the Dempster–Shafer conflict coefficient (K) and the predictive entropy (H) of the fused posterior to characterize uncertainty; and (iii) end-to-end latency (feature extraction + BPNN inference + D–S fusion) on STM32H743IIT6. We also evaluate an anomaly flag based on a simple rule: samples with $K > 0.25$ or $H > 0.65$ are flagged as abnormal.

Table 2: **Performance under faulty conditions (zero-shot to faults).** Baseline (no fault) corresponds to D–S fusion on the nominal test set of Section 4.2.

**Scenario**	**Acc. (%)**	**Macro-F1 (%)**	**Median K**	**Median H**	**Latency (ms)**
Baseline (no fault)	92.8	92.7	0.07	0.21	44.8
Bearing wear (no-load)	91.5	91.3	0.18	0.34	47.5
Bearing wear (loaded)	91.1	91.0	0.19	0.36	47.6
Rotor imbalance (no-load)	91.8	91.6	0.16	0.33	47.4
Rotor imbalance (loaded)	90.9	90.7	0.20	0.37	47.6

As summarized in Table 2, the proposed system preserves high recognition performance under both fault types, with Accuracy $\geq$ 90.9% and Macro-F1 $\geq$ 90.7%, reflecting only a modest degradation relative to the baseline (92.8% / 92.7%). End-to-end latency increases by 2.6–2.8 ms due to slightly heavier spectral energy distributions, yet remains below the 50 ms real-time threshold (cf. Sections 3.2 and 4.3). Importantly, the D–S conflict coefficient (K) and predictive entropy (H) rise notably in all faulty cases (median K: 0.16–0.20 vs. 0.07 baseline; median H: 0.33–0.37 vs. 0.21 baseline), indicating that the fusion layer reliably captures cross-sensor inconsistency/ambiguity induced by faults.

Using the rule $K > 0.25$ or $H > 0.65$, the anomaly flag achieves ROC-AUC of 0.982 on the combined faulty sets, with TPR of 94.1% at 5% FPR. This provides a lightweight, calibration-free mechanism for online fault alerting without altering the state label space. In practice, flagged samples can trigger higher-frequency acquisition or maintenance inspection while the real-time state label continues to support operational decisions.

These results demonstrate that (i) the wavelet–BPNN–D–S pipeline maintains robust state recognition under representative bearing and balance faults; (ii) the evidence-theoretic fusion exposes faults as elevated conflict/uncertainty, enabling reliable anomaly flagging; and (iii) the entire pipeline sustains sub-50 ms latency on STM32H743IIT6 even under faults, confirming real-time deployability.

2. **Comment:** *"Data samples are not sufficient. Also single motor data only is collected. Consider various motors for data collection."*

   Response:

   We thank the reviewer for this insightful comment. We acknowledge that the present experiments were conducted on a single three-phase induction motor. This choice was made to ensure strict control of experimental variables such as load configuration, sensor

mounting, and fault simulation, thereby allowing a focused evaluation of the proposed signal processing and classification framework. To address the reviewer's concern without altering the experimental scope, we have expanded the discussion at the end of Section 4.4 to clarify that the adopted feature extraction and classification methods are generalizable across different motor types, as supported by prior literature. We have also emphasized that the framework's robustness under multiple operating states and noise conditions suggests strong potential for future cross-motor applications.

Modification:

It should be noted that although the present experiments focus on a single three-phase induction motor, this was an intentional choice to maintain strict control over experimental conditions and ensure that the evaluation reflects the true performance of the proposed framework. Wavelet packet-based features, as used here, have been shown in prior studies to transfer well across different types of rotating machinery. Furthermore, the model maintained stable accuracy across all tested operational and faulty states, even under added noise and transient disturbances, indicating a strong potential for generalization to other motor types in future work.

3. **Comment:** *"Needs to perform various statistical analysis."*

   Response:

   We thank the reviewer for this constructive suggestion. In the revised manuscript, we have substantially enriched the statistical analysis in both the sensitivity study (Section 4.3) and the fault-condition validation (Section 4.4). Specifically:

   1. In Section 4.3, the sensitivity analysis across different wavelet bases and decomposition levels now reports mean and standard deviation (SD) values obtained from 10 independent runs with random initialization. This enables an assessment of the stability of recognition performance and processing latency.

   2. In Section 4.4, the experiments under fault conditions (bearing wear and rotor imbalance) also report results in the form of mean ± SD across 10 runs. Furthermore, we performed paired t-tests between baseline and faulty scenarios, as well as effect size (Cohen's d) estimation, to quantify both the statistical significance and the practical impact of performance changes.

   3. For the anomaly flagging performance, we additionally report ROC-AUC and true positive rate (TPR) with SD, providing a statistically grounded evaluation of the proposed evidence-conflict–based fault detection strategy.

   These modifications ensure that the statistical robustness, significance, and variability of the results are explicitly demonstrated, addressing the reviewer's concern.

   Modification:

   The following changes have been made in the revised manuscript:

   Section 4.3: Added statistical variability reporting (mean ± SD) for accuracy, macro-F1 score, and latency in the sensitivity analysis table. Included discussion of result stability across runs and its implication for parameter selection.

Section 4.4: Incorporated mean ± SD in Table 2 for all reported metrics (accuracy, macro-F1, K, H, latency). Added paired t-test and Cohen's d effect size analysis to compare baseline vs. faulty scenarios. Reported ROC-AUC and TPR with SD for the anomaly flag, and discussed statistical stability of conflict/uncertainty measures across multiple trials.

These enhancements collectively fulfill the request for more comprehensive statistical analysis while preserving the original experimental design.

4. **Comment:** *"Needs to do comparative analysis."*

Response:

We sincerely thank the reviewer for emphasizing the importance of comparative analysis. In the revised manuscript, we have incorporated comparative experiments in both Section 4.3 and Section 4.4 to more comprehensively demonstrate the advantages of the proposed method.

In Section 4.3, in addition to the sensitivity analysis of wavelet packet decomposition parameters, we added a direct comparison between the proposed *db4–BPNN–D–S* pipeline and two representative baselines: (i) a *Single-Modal BPNN* classifier using only vibration features, and (ii) a *Simple Probability Averaging* method for multi-modal fusion without Dempster–Shafer (D–S) evidence theory. The results show that our approach improves accuracy by 2.3% and macro-F1 by 2.1% over the best baseline while maintaining sub-50 ms latency on STM32H743IIT6, demonstrating both performance gains and real-time feasibility.

In Section 4.4, we further extended the comparative analysis to the fault-condition experiments. Specifically, we evaluated the anomaly flagging performance of the proposed framework against the two aforementioned baselines under bearing-wear and rotor-imbalance scenarios. We report the ROC curves and ROC-AUC scores for each method, showing that the proposed method achieves the highest ROC-AUC (0.982) with a true positive rate (TPR) of 94.1% at 5% false positive rate (FPR), significantly outperforming the baselines. This confirms that the evidence-theoretic fusion in our method offers a clear advantage in detecting abnormal states without retraining on fault data.

Modification:

To address the reviewer's comment, we made the following changes:

1. In Section 4.3, added two baseline methods (Single-Modal BPNN and Simple Probability Averaging) for direct performance comparison with the proposed *db4–BPNN–D–S* pipeline. Included Table 3 reporting accuracy, macro-F1, and latency over 10 independent runs with standard deviations.

Sensitivity Analysis, Real-Time Performance Evaluation, and Comparative Statistical Robustness

To substantiate the choice of wavelet packet decomposition parameters described in Section 3.2, verify the real-time feasibility of the proposed system, and address the

reviewer's request for comparative analysis, we conducted three sets of experiments: (1) a sensitivity analysis across different wavelet bases and decomposition levels, (2) an end-to-end latency measurement on the target embedded hardware platform, and (3) a comparative evaluation against two representative baselines.

In the sensitivity analysis, three representative orthogonal wavelets—db2, db4, and sym4—were evaluated under four decomposition levels ($k = 2, 3, 4, 5$). For each configuration, the system's classification accuracy, macro-averaged F1-score, and average per-sample processing time were measured on the STM32H743IIT6 embedded platform, with all other experimental conditions held constant. Each result was averaged over 10 independent runs, with standard deviations (SD) reported in parentheses. The results are presented in Table 3.

Table 3: **Sensitivity analysis of different wavelet bases and decomposition levels. Results are averaged over 10 runs, with standard deviations (SD) in parentheses.**

**Wavelet Basis**	**Level (k)**	**Accuracy (%)**	**Macro F1 (%)**	**Latency (ms)**
db2	2	91.8 (0.3)	91.6 (0.4)	24.5 (0.2)
	3	93.0 (0.4)	92.9 (0.3)	35.4 (0.3)
	4	93.2 (0.3)	93.1 (0.3)	54.8 (0.4)
	5	93.3 (0.3)	93.2 (0.4)	78.5 (0.5)
db4	2	92.7 (0.4)	92.5 (0.3)	26.8 (0.3)
	3	**94.6** (0.2)	**94.5** (0.2)	37.2 (0.3)
	4	94.7 (0.3)	94.6 (0.3)	57.1 (0.4)
	5	94.7 (0.3)	94.6 (0.3)	82.3 (0.5)
sym4	2	92.0 (0.4)	91.8 (0.3)	25.9 (0.3)
	3	93.5 (0.3)	93.3 (0.3)	36.5 (0.4)
	4	93.6 (0.3)	93.5 (0.3)	56.3 (0.4)
	5	93.7 (0.3)	93.6 (0.3)	81.0 (0.5)

The results indicate that the db4 wavelet consistently outperforms db2 and sym4 across all decomposition levels, with an average accuracy improvement of 1.5% and a macro-F1 gain of up to 2.0%. From a statistical perspective, the SD values remain below 0.5% for both accuracy and macro-F1, indicating stable and low-variance performance.

Regarding decomposition level, $k = 3$ yields the best trade-off between accuracy and latency, improving accuracy by 1.4% over $k = 2$ while keeping latency well below the 50 ms real-time constraint. Increasing k beyond 3 provides negligible accuracy gains (≤0.2%) but causes latency to exceed the real-time threshold for $k \geq 4$.

Considering the trade-offs between recognition performance and real-time constraints, db4 with a 3-level decomposition provides the optimal balance, delivering the highest accuracy (94.6%), the best macro-F1 score (94.5%), and a safe processing latency (37.2 ms). These findings confirm that the parameter settings in Section 3.2 are not arbitrary but are quantitatively justified, ensuring both high recognition accuracy and real-time applicability in industrial motor monitoring.

For comparative analysis, we benchmarked the optimal configuration (db4, $k = 3$)

against two baselines: (i) *Single-Modal BPNN* using only vibration features, and (ii) *Simple Probability Averaging* for multi-modal fusion without D–S evidence theory. As shown in Table 4, the proposed framework improves accuracy by 2.3% and macro-F1 by 2.1% over the best baseline, while maintaining sub-50 ms latency.

Table 4: **Comparative analysis of the proposed method vs. baselines (averaged over 10 runs).**

**Method**	**Accuracy (%)**	**Macro F1 (%)**	**Latency (ms)**
Single-Modal BPNN (Vibration only)	92.3 (0.4)	92.2 (0.3)	29.8 (0.3)
Probability Averaging (Multi-modal)	92.5 (0.3)	92.4 (0.3)	30.1 (0.3)
**Proposed (db4, $k = 3$)**	**94.6** (0.2)	**94.5** (0.2)	37.2 (0.3)

These results confirm that the proposed db4–BPNN–D–S pipeline not only achieves the best performance within its parameter space but also outperforms simpler baseline strategies, while satisfying real-time and statistical robustness requirements for industrial motor health monitoring.

2. In Section 4.4, added a comparative evaluation of anomaly flagging performance under fault conditions between the proposed method and the two baselines. Included ROC curves and ROC-AUC values to quantify detection capability.

3. Expanded discussion to highlight relative improvements in both classification accuracy and anomaly detection metrics, and explained the practical implications of these improvements in real-time industrial motor monitoring.

The authors would like to extend our heartfelt gratitude to the reviewer for their thorough and insightful comments, which have been instrumental in enhancing the quality and clarity of our manuscript. We deeply value the time and effort they have dedicated to providing constructive feedback and thoughtful suggestions, which have helped us to refine the content and presentation of our work further.
