# Supplementary material for: Real-time motor operating state recognition via multi-sensor fusion: A wavelet–neural–evidence framework for industrial condition monitoring
Source: PLoS One. 2025 Nov 7;20(11):e0335360. doi: 10.1371/journal.pone.0335360 (PMC12594399; doi:10.1371/journal.pone.0335360)
Supplement: Attachment — Submitted filename: Response_to_Reviewers_auresp_2.pdf [file pone.0335360.s003.pdf]

Response to Reviewer No. 3:

PONE-D-25-33127R1

"Real-Time Motor Operating State Recognition via Multi-Sensor Fusion: A Wavelet–Neural–Evidence Framework for Industrial Condition Monitoring"

We greatly appreciate the reviewers' efforts in evaluating our manuscript. Based on the feedback received, we have revised our document to improve both the accuracy and clarity. To assist the reviewers in identifying these changes, we have highlighted the sections revised in response to **Reviewer 3's comments** in **red**. Thank you for the constructive insights that have helped enhance our work.

1. **Comment 1:** *"You state that continuous recordings were windowed into 1024-sample segments with 50% overlap and then split 60/20/20. Please make explicit that the split is done at the recording/session (or subject) level—not at the window level—so that no windows from the same 10-minute session appear in both training and test sets."*

   Response: We appreciate the suggestion and confirm that the 60/20/20 split is performed at the *recording/session level*, not at the window level. Specifically, each 10-minute continuous recording (per state and per acquisition round) is treated as an indivisible unit for data partitioning to avoid temporal leakage from overlapped windows across sets. No window from a given session appears in more than one split (train/validation/test). To further guard against leakage, all hyperparameters were tuned exclusively on the validation set, and the test set remained untouched until final evaluation. As a robustness check, we also repeated the evaluation using a session-grouped (blocked) split; performance differences were within ±0.3% for Accuracy and Macro-F1.

   Modification (Section 4.1, end of "Dataset composition and labeling"): "To avoid any temporal leakage from overlapped windows, the 60%/20%/20% partition is conducted at the *recording/session* level: each 10-minute continuous recording is assigned wholly to either train, validation, or test, so that no window from the same session appears across different splits. Hyperparameters are tuned only on the validation set, and the test set remains untouched until the final evaluation. A session-grouped re-evaluation yielded Accuracy and Macro-F1 within ±0.3% of the main results, confirming robustness."

2. **Comment 2:** *"You note dataset availability, but there is no link to the processing/training code and the embedded inference code. Public code greatly improves reproducibility and speeds up adoption."*

   Response: We thank the reviewer for this important suggestion. To ensure reproducibility and facilitate adoption, we have provided all relevant code and firmware as a supplementary file. This supplementary material (S1) accompanies the revised submission and will remain permanently accessible with the publication.

   Modification (Section 6, Supporting information): "**Code and Firmware Availability.** The supporting file (S1.pdf) contains the paper code. This package ensures full reproducibility of the results and will remain permanently accessible with the article."

3. **Comment 3:** *"Discuss complex and overcomplete wavelets (e.g., dual-tree complex, rational-dilation, tunable Q-factor) as extensions; cite the listed works."*

   Response: We thank the reviewer for this constructive suggestion. While our current design prioritizes orthogonal real-valued WPT (db4, k=3) for its balance of accuracy, latency, and memory footprint on embedded hardware, we agree that complex and overcomplete wavelet families offer valuable extensions for early-fault detection. Specifically: (i) the dual-tree complex wavelet transform (DTCWT) provides approximate shift-invariance and directional selectivity, (ii) rational-dilation wavelets enable flexible time–frequency tiling, and (iii) tunable Q-factor wavelets (TQWT) are well suited for oscillatory transient capture. These properties can improve robustness against phase shifts, enhance separation of closely spaced spectral components, and strengthen weak-fault signature recognition, albeit at the cost of higher computational complexity. We have therefore added a short Discussion paragraph highlighting these extensions and citing the recommended references.

   Modification: (added at the end of Section 3.1 or as a brief Discussion paragraph before the Conclusion)

   "*Discussion—Complex and Overcomplete Wavelets as Extensions.* Beyond the orthogonal WPT adopted here for embedded efficiency, complex and overcomplete wavelet families are promising for early-fault regimes. The dual-tree complex wavelet transform (DTCWT) provides approximate shift-invariance and directional selectivity that may reduce sensitivity to phase jitter and improve feature stability. Rational-dilation constructions offer flexible time–frequency partitioning for separating closely spaced components, while tunable Q-factor wavelets (TQWT) can capture weak oscillatory transients more effectively. These approaches inevitably increase computational demand, but hybrid strategies—such as combining complex/overcomplete front-ends with lightweight classifiers and selective channel pruning—could help achieve real-time feasibility. Representative methodologies include 26-27."

The authors would like to express our sincere appreciation to the referee for the constructive comments and the efforts provided in helping us to improve the quality and presentation of the paper further.
